# Economic Burden of Severe Hypoglycemia Among Patients With Diabetes Mellitus

**DOI:** 10.7759/cureus.31889

**Published:** 2022-11-25

**Authors:** Afsar Ahammed, AHM Aktaruzzaman, Abu J Gaffar, Faria Afsana, Ahmed S Mir, Lutful Kabir, Shahjada Selim, Md. F Pathan

**Affiliations:** 1 Department of Endocrinology, National Institute of Traumatology and Orthopaedic Rehabilitation (NITOR), Dhaka, BGD; 2 Department of Endocrinology, Rajshahi Medical College and Hospital (RMCH), Rajshahi, BGD; 3 Department of Endocrinology, Naogaon Medical College and Hospital, Naogaon, BGD; 4 Department of Endocrinology, Bangladesh Institute of Research and Rehabilitation in Diabetes (BIRDEM), Dhaka, BGD; 5 Department of Endocrinology, Bangladesh Institute of Health Sciences, Dhaka, BGD; 6 Department of Endocrinology, Rangpur Medical College and Hospital, Rangpur, BGD; 7 Department of Endocrinology, Bangabandhu Sheikh Mujib Medical University Hospital (BSMMU), Dhaka, BGD

**Keywords:** severe hypoglycemia, productivity of life, indirect cost, direct cost, cost-of-illness

## Abstract

Background: Bangladesh is anticipated to have the eighth-highest number of diabetic patients within the next 15 years. Approximately one-fifth of adult diabetes patients reside in Southeast Asian nations. This study aimed to find out the economic burden of extreme hypoglycemia on diabetic sufferers in Bangladesh.

Methods: A cross-sectional study was carried out amongst 164 Type 2 Diabetes sufferers admitted due to extreme hypoglycemia within 15 months at BIRDEM in Dhaka to decide if they have the impact of extreme hypoglycemia on the cost of illness. The cost was once expressed in BDT.

Results: Direct medical cost (37058) and direct non-medical cost (5261) was estimated during the study. Among the direct medical cost, hospital cost was 17735, physician cost was 5745, nonmedical transport cost was 1802, and attendant cost was 3459. The total cost was 48743 BDT (€617) for each severe hypoglycemic event leading to hospitalization, and 6.4244 BDT (€82.4) would be the indirect cost of reduced productivity from spending 5.8 days (46.4 hours) in the hospital.

Conclusion: The analysis indicates that hypoglycemia has a significant negative influence on the cost and reduces the work output of diabetics.

## Introduction

Non-communicable diseases (NCDs) have been more prevalent in Bangladesh over the past few decades, and diabetes is one of the most concerning NCDs in terms of public health. Bangladesh is anticipated to have the eighth-highest number of diabetic patients within the next 15 years. Adult diabetes patients comprise about one-fifth of the population in Southeast Asian countries. [1]. The nation is increasingly susceptible to diabetes due to fast urbanization, the country's economic growth, dietary patterns, sedentary lifestyles, rising life expectancy, high consumption of salt and foods high in chemical energy, and even genetic predisposition. Diabetes treatment expenses can have a significant negative financial impact on households and entire nations [2]. There were about 55,703 diabetics in 2016, with a projected 26,41,000 OPD visits. The estimated total annual cost of diagnosed diabetes was around USD 217.71 million in Bangladesh. Since diabetes is a chronic condition, the economic impact is highly complex and challenging to quantify [3]. Personal diet, physical activity, and appropriate medicine are essential for managing diabetes; however, among Bangladeshis, adequate diabetes care is uncommon due to a lack of time, a lack of venues that are suited for walking or exercising, and even a misunderstanding of those activities [4].

Diabetes-related medical costs are rising at a greater frequency around the globe, placing a tremendous strain on sufferers [5]. One of the most significant side effects of diabetes medication is hypoglycemia. Identification and prevention of hypoglycemia can lessen the impact of diabetes by preventing complications from hypoglycemia. Severe untreated hypoglycemia can have a major financial and personal impact [6]. Patients with diabetes mellitus who have severe hypoglycemia may experience substantial cognitive dysfunction as well as deadly vascular events such as myocardial infarction, stroke, and cardiac arrhythmias, which have a significant financial impact [7]. This study aimed to assess the economic burden of severe hypoglycemia on diabetic patients in Bangladesh.

## Materials and methods

A total of 164 Bangladeshi T2DM patients admitted to the specialist hospital BIRDEM, Shahbag, Dhaka, due to severe hypoglycemia, participated in this cross-sectional study. The sample was taken purposively. The period was 15-month. The inclusion criteria comprise all socioeconomic strata's diabetic patients above the age of 18. Patients who didn't want to participate in the study, patients with hypoglycemia who weren't diabetic, pregnant women, and those under the age of 18 were excluded. Ethical clearance was obtained from the Institutional Review Board of BIRDEM vide letter no. of "BADAS-ERC/EC/15/00257". The purpose of the study was explained to each patient and relative in detail.

The data was collected in a pre-formed standard printed data collection form after taking written informed consent from the patient, but in the case of a semiconscious or unconscious patient, it was taken from the patient’s relative or after full recovery of the patient. Data collection forms were filled in at the hospital at the bedside every day. After primary selection, a detailed clinical history severity of hypoglycemia was assessed. The blood glucose level at the time of hypoglycemia was collected. In the BIRDEM Hospital, the cost of hypoglycemia in diabetic patients is estimated using a cost-of-illness methodology. The study's technique is incidence-based, which takes into consideration all newly diagnosed cases of hypoglycemia in a given year.

The costs of different hypoglycemia occurrences are calculated using a bottom-up approach. Both the direct and indirect costs of hypoglycemia were taken into account. Indirect costs comprised lost productivity as a result of hypoglycemic occurrences, while direct costs included expenses for medically treating hypoglycemia. Three fundamental assumptions were made for determining the cost of lost productivity: 1) A person having a hypoglycemic episode wouldn't come to work that day; 2) If the incident happened at night, productivity would be lost for half a day; and 3) There would be no productivity loss if the event happened after work but before going to sleep. All of the data were reviewed after collection, edited, and through the aid of SPSS Inc. Released 2008. SPSS Statistics for Windows, Version 17.0. Chicago: SPSS Inc. the data was inserted into the computer. Each respondent has been given his own code, and each code was entered separately. Due to analysis, data has been transformed, computed, and recorded by giving the mark to the respondent then the mean value is calculated. Where necessary, chi-square and t-tests were performed. The 95% confidence interval was calculated, and the significance level was set at p = 0.05.

## Results

An overall of 182 patients had been enrolled in the study. Of them, seven patients had spontaneous hypoglycemia that used to be now not verified as diabetic, and 11 patients refused to be involved in the study. Data from the ultimate 164 patients have been gathered and analyzed for the study. Among them, 78 were male (47.6%), and 86 were female (52.4%) (Table 1). The maximum number of patients, 72 (43.90%), belonged between 41 and 60 years. These finds out about determined no statistically extensive distinction between the incidences of hypoglycemia in demographic distribution. However, the incidence used to be a lot higher, 96 (58.5%) in less educated persons than those who are quite educated. The relationship of BMI with extreme hypoglycemia was once evaluated. It was found that the observed majority of 81 (49.40%) hypoglycemia patients had a BMI between 18-22.9 kg/m^2^, i.e., average weight in accordance with the Asian category. This suggests that perfect hypoglycemic 98 (59.70%) patients had a period of diabetes >5-20 years. According to glycemic status, most of them have been 102 (62.20%) in the uncontrolled group (HbA1c ≥ 7%). Patients who used insulin with a syringe had a higher rate of 84 (69.40%) of hypoglycemic activities than those who used a pen 37 (30.60%). Mixed insulin users (both premixed and break-up mixed) had the absolute best incidence of 96 (79.30%), while only long-acting insulin users had the lowest rate of five (4.10%) of hypoglycemic events. Among the extreme hypoglycemia, significantly, 102 (62.20%) recovered from hypoglycemia by using IV glucose, and 62 (37.80%) recovered via oral glucose. Severe hypoglycemia's economic burden is divided into direct costs (Table 2, 3) and indirect costs (Table 4, 5). Table 6 shows the mean of total expenditure in controlled and uncontrolled diabetes mellitus patients (N=164).

**Table 1:** Demographic and clinical characteristics

Variable	Domain		Number	Percentage
Age group (years)	>18-40		15	9.10%
	41-60		72	43.90%
	61-80		66	40.30%
	>80		11	6.70%
Gender	Male		78	47.60%
	Female		86	52.40%
Education	less than HSC		96	58.50%
	≥ HSC		68	41.50%
Body Mass Index (Kg/m2)	< 18		10	6.10%
	18-22.9		81	49.40%
	≥23		73	44.50%
Duration of T2DM (years)		<5	39	23.80%
		5-20	98	59.70%
		>20	27	16.50%
HbA1c (%)		Controlled (<7%)	62	37.80%
		Uncontrolled (≥7)	102	62.20%
Insulin Regime		Only Short Acting	14	11.60%
		Only Long Acting	6	5.00%
		Premixed	64	52.90%
		Split Mixed	32	26.40%
		Basal Bolus	5	4.10%
Injection Device		Syringe	84	69.40%
		Pen	37	30.60%
Recovery Method		Intravenous Glucose	102	62.20%
		Oral Glucose	62	37.80%

**Table 2:** Direct medical expenditure among the respondents (N=164)

Domain	Expenditure	Mean ±SD	Median	Range (Min-max)
Hospital cost	Admission Fee	288 ± 137	200	200 – 500
	Bed Cost	12955 ± 14782	8400	1400 – 114400
	Service Charge	1357 ± 1213	990	210 – 7982
	Medicine Cost	3317 ± 6422	2000	500 – 50000
	Others	6067 ± 7862	3000	200 – 15000
	Total Hospital cost	17735 ± 18523	11845	2550 – 130130
Physician/ Consultant Cost	Consultant Visit	3056 ± 2727	2500	100 – 25500
	Junior Doctor Visit	1763 ± 1244	1500	300 – 7800
	Referral Visit	1342 ± 1033	1000	400 – 8000
	Nutritionist Visit	200 ± 0	200	200 – 200
	Others	17045 ± 18553	11850	1000 – 43480
	Total Physician/ Consultant Cost	5745 ± 4306	4400	900 – 24500
Investigation Cost	Blood Test	9220 ± 5769	7675	600 – 45350
	Strip Test	1182 ± 1091	900	50 – 10500
	Urine Test	522 ± 451	550	150 – 2050
	Imaging Test	2089 ± 1972	1300	200 – 10500
	Others	3282 ± 7917	800	150 – 43455
	Total Investigation Cost	13578 ± 9850	11565	900 – 74500

**Table 3:** Direct non-medical expenditure – Attendant cost among the respondents (N=164)

Expenditure	Mean ±SD	Median	Range (Min-max)
Food cost	3037 ± 2982	2150	300 – 19000
Transport cost	423 ± 274	400	50 – 1800
Attendant cost	3459 ± 3213	2400	300 – 20800

**Table 4:** Indirect expenditure – Productivity loss cost among the respondents (N=164)

Expenditure	Per Day Income Loss (Mean ±SD)	Per Day Income Loss (Median)	Range (Min-max)
Patient	7462 ±15221	1000	00 – 90000
Care Giver	6399 ±9389	1100	00 –80000
Indirect Productivity Loss Cost	8428 ± 16846	4500	00 – 158000

**Table 5:** Mean of total productivity loss in days for controlled and uncontrolled diabetes mellitus patients (N=164)

Expenditure	N=164	Mean ±SD (Days)	Median (Days)	Range (Min-max)	p Value
Controlled (HbA1c<7%)	62	5.27±3.667	4	1-16	.217
Uncontrolled (HbA1c≥7%)	102	6.06±4.088	4	1-22	
Total	164	5.76 ± 3.94	5	1-22	

**Table 6:** Mean of total expenditure in controlled and uncontrolled diabetes mellitus patients (N=164)

Expenditure	Mean ±SD	Median	Range (Min-max)	p-value
Controlled (HbA1c<7%)	42608 ± 34049	34870	8310 – 220298	0.150
Uncontrolled (HbA1c≥7%)	52472 ± 46718	38602	8210 – 372380	
Total Cost	48743 ± 42536	36655	8210 – 372380	

**Figure 1:**
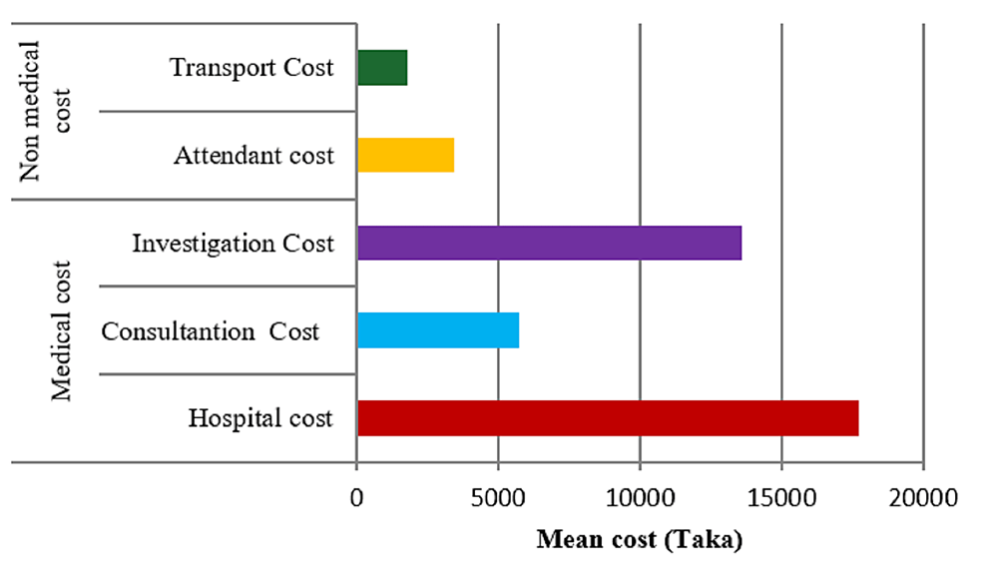
Direct medical and non-medical mean cost in the study population (N=164)

**Figure 2:**
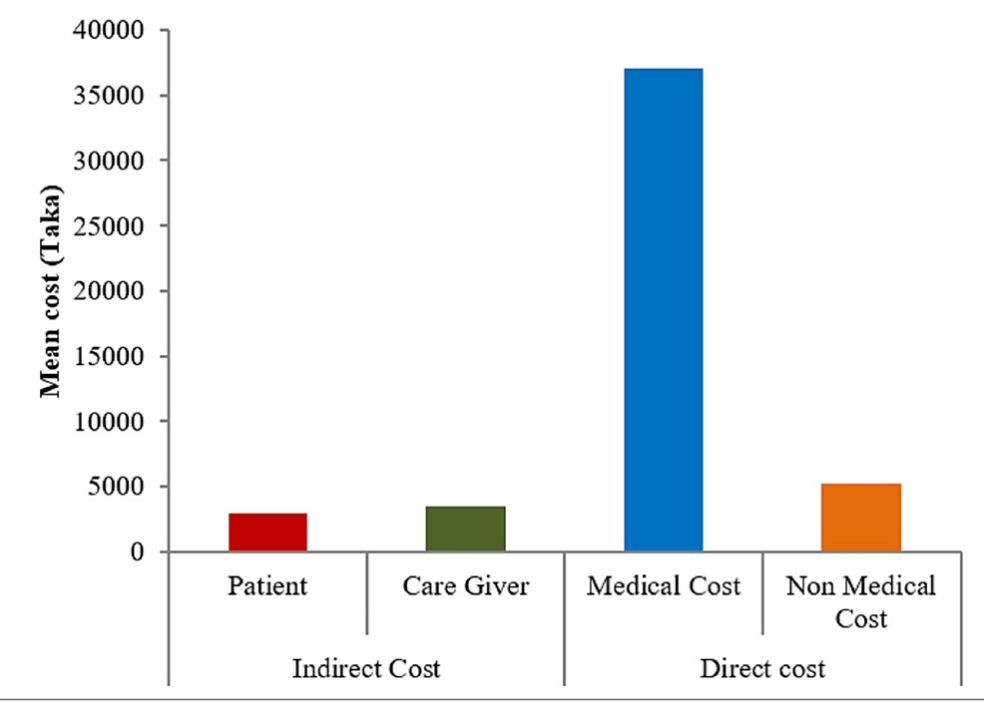
Direct and indirect mean cost in the study population (N=164)

The direct value consists of medical and non-medical costs. Medical cost is categorized into hospital, consultant, investigation cost, non-medical costs, transport, and the attendant cost. Indirect cost was once productiveness loss for each affected person and attendant. In our study, mean hospital costs included admission cost (mean = 288), bed cost (mean = 12955), service charge (mean = 1357), medicine cost (mean = 3317), others, including oxygen, nebulization, etc. (mean = 6067), and total hospital cost mean = 17735 (Figure 1). Consultation cost includes senior consultant cost (mean = 3056), junior doctor cost (mean = 1763), referral cost (mean = 1342), nutritionist cost (mean = 200), and other costs, including eye, ENT (ear, nose, tongue), surgery, dental procedure, etc. (mean = 17045) and total consultation cost was mean = 5745. Among the investigation cost, blood cost (mean = 9220), strip cost (mean = 1182), urine cost (mean = 522), imagine cost (mean 2089), others, including histopathology, biopsy, fine needle aspiration cytology (FNAC), etc., cost (mean = 3282), and total investigation cost was mean = 13578. On the other hand, non-medical transport costs: ambulance cost (mean = 3394), bus cost (mean = 706), private car cost (mean = 1494), CNG (compressed natural gas) cost (mean = 290), rickshaw cost (mean = 60), others transport cost train, launch, etc. (mean = 1467), and total transport cost mean = 1802. Non-medical attendant total cost mean was 3459 and attendant food cost (mean = 3037), and attendant transport cost (mean = 423) (Figure 2). Indirect productivity loss of patient cost (mean = 2958) and attendant productivity cost (mean = 3472) and mean cost of total productivity loss was BDT 6424. Indirect productivity means day loss was 5.8 days (46.4 hours). So, per admission, due to severe hypoglycemia mean total cost was 48743 (£617).

## Discussion

Diabetes mellitus significantly raises healthcare and societal expenditures. The primary problem with diabetes is the surge in direct costs, particularly when it comes to treating acute and long-term problems that frequently require hospital care. Among acute complications, severe hypoglycemia (SH) is the most common [8].

In this study, the value-added cost of hypoglycemia incidents was not factored into the calculation. Even though there had been no known deaths within the study, hypoglycemia-related mortality was also excluded. This study shows that hypoglycemia episodes in diabetic individuals are linked to high costs and decreased productivity. The mean total cost was 48743 BDT, or (€ 617). In addition, 5.8 days (46.4 hours) in the hospital would cost 6424 BDT (€82.4) in indirect lost productivity costs. A French study estimated the total hospital cost of a stay for hypoglycemia to be FF14,000 (US$2100). This study also showed that 10,800 out of 40,000 events of severe hypoglycemia led to hospitalization [9]. In our study, the average length of stay in the hospital was 6.6 days for FF14,000. This is similar to the average length of stay for patients hospitalized because of diabetes under the diagnosis-related groups (DRG) in Sweden, equating to an average cost of €2806.8 for each severe event leading to hospitalization. In addition, the indirect cost of lost productivity from spending 6.6 days (52.8 h) in the hospital would be €1110.6 [10].

It was observed that uncontrolled patients' costs for severe hypoglycemia per event were greater than those of controlled patients' costs (mean, 52472), but no significant correlation was discovered (p-value 0.150). The mean cost of hypoglycemia in our study is 86.82% of direct cost and 13.18% of indirect cost. One study was reported in Spain [11] in which the mean cost of hypoglycemia was 65.4% of direct cost and 34.6% of indirect cost. Few empirical studies have assessed the cost of hypoglycemia. Two European surveys examined both direct and indirect costs incurred in the management and follow-up of severe hypoglycemia events [12,13]. Leese et al. (2003) estimated the cost of severe hypoglycemia to be about £380 per event [14]. A German study estimated the cost of severe hypoglycemia to be USD44,338/per 100,000 inhabitants [15]. The total cost of hypoglycemia in patients with Type 2 diabetes in Sweden was estimated to be approximately €4,250,000 (€14.1 per patient with Type 2 diabetes) [16]. The cost can be avoidable by minimizing hypoglycemic episodes, and a minimum reduction of frequent hypoglycemic episodes can cause a significant reduction in economic burden [17].

## Conclusions

Hypoglycemia is once in a while stated as a 'safety issue; however, a more comprehensive research focus, such as evaluation of the consequence of hypoglycemia on quality of existence as a co-primary final stage alongside glycemia control, is requisite to apprehend and quantify the unsafe personal, social and economic implications of hypoglycemia on people with diabetes. This finding is essential for ordaining the value of treatment in economic evaluations and exposes massive scope for improvement in health status for people suffering from hypoglycemia. It suspects an effective humanistic and economic impact on the growing rapidity and recurrence of hypoglycemia. The finding also amplifies the necessity to make sure that people with diabetes are aware of hypoglycemia, and efforts should be made to minimize the incidence of hypoglycemia and decrease the load of treatment costs while striving to gain reasonable control.
